# Localized Photoactuation of Polymer Pens for Nanolithography

**DOI:** 10.3390/molecules28031171

**Published:** 2023-01-25

**Authors:** Zhongjie Huang, Shaopeng Li, Jiaqi Zhang, Huan Pang, Andrey Ivankin, Yuhuang Wang

**Affiliations:** 1 State Key Laboratory for Modification of Chemical Fibers and Polymer Materials, College of Materials Science and Engineering, Donghua University, Shanghai 201620, China; 2 School of Chemistry and Chemical Engineering, Yangzhou University, Yangzhou 225009, China; 3 TERA-print, LLC, 8045 Lamon Ave, Suite 330, Skokie, IL 60077, USA; 4 Department of Chemistry and Biochemistry, University of Maryland, College Park, MD 20742, USA; 5 Maryland NanoCenter, University of Maryland, College Park, MD 20742, USA

**Keywords:** photoactuation, digital micromirror device, PDMS, nanolithography, nanocomposite, carbon nanotube

## Abstract

Localized actuation is an important goal of nanotechnology broadly impacting applications such as programmable materials, soft robotics, and nanolithography. Despite significant recent advances, actuation with high temporal and spatial resolution remains challenging to achieve. Herein, we demonstrate strongly localized photoactuation of polymer pens made of polydimethylsiloxane (PDMS) and surface-functionalized short carbon nanotubes based on a fundamental understanding of the nanocomposite chemistry and device innovations in directing intense light with digital micromirrors to microscale domains. We show that local illumination can drive a small group of pens (3 × 3 over 170 μm × 170 μm) within a massively two-dimensional array to attain an out-of-plane motion by more than 7 μm for active molecular printing. The observed effect marks a striking three-order-of-magnitude improvement over the state of the art and suggests new opportunities for active actuation.

## 1. Introduction

Actuators, which turn control signals to mechanical actions, are key drivers in advancing modern technology and shaping our future. In recent years, enormous efforts have been put into the development of soft actuators [1,2,3,4,5] made of stimuli-responsive polymers, hydrogels, liquid metals, phase-change materials, and composites, [6,7,8,9,10,11,12,13] due to their flexibility, adaptability, biocompatibility, and multi-functionality. Substantial progress has been made on the deformation of the macroscopic material/system under external stimuli. Nevertheless, local actuation with high spatiotemporal precision, resolution, and selectivity remains one of the exciting challenges in nanotechnology owing to the wealth of fundamental questions and ample potential applications in programmable materials, micro/nano robotics, haptic rendering, and precision medicine, [13,14,15,16,17,18,19,20] towards which efforts are needed in both material science and device engineering. It is essential to comprehend the microscopic condition of the functional components in the composites, including the microarchitecture, interface chemistry, and assembly/embedding strategy. In addition, there require approaches to deliver site-specific external stimuli with high spatiotemporal resolution in a programmable manner. Recently there have been encouraging advances in spatially selective addressing and multiplexing in various actuating systems; however, most of the progress depends on the integration of sophisticated physical systems, such as arrays of microelectrodes, microcoils, and cavities [21,22,23,24].

Three-dimensionally crosslinked siloxane elastomer polydimethylsiloxane (PDMS) has become the material of choice for microfluidic and microelectromechanical systems, flexible electronics, bioengineering, soft robotics, and soft lithography [25,26,27,28,29,30,31,32]. A wide variety of fillers have been incorporated into the PDMS matrix for adding intriguing optical, thermal, electrical, magnetic, or mechanical properties, [33,34] but homogenous dispersion of the fillers in the elastomer matrix remains challenging, [35] which impedes the overall performance and high-resolution actuation. Photoactuation represents an elegant and attractive strategy for triggering local mechanical work with high spatiotemporal precision and resolution, due to its intrinsic ability of remote control, wireless access, and spatial selectivity. PDMS is blessed with excellent optical transparency at 200–1100 nm, which allows the integrated functional fillers to tailor the optical characteristics. Besides, PDMS is known for its facileness in producing various microstructures, which can provide rich deformation modes with designed anisotropic architectures.

Cantilever-free polymer pens are powerful duplication tools for nanolithography; however, all the pens in the same array define identical duplicates instead of diverse features [36]. The capability to control individual pens is a long-standing goal in this field and will translate this tool to a general nanofabrication platform. Previously, we demonstrated that microscopic light can be used to actuate a group of pens (~3000) in a massive array for molecular printing [37,38]. However, the actuation due to photothermal effect becomes less significant when the illumination area decreases to microscale (which covers fewer pens) [39]. The key challenge is the need for approaches to achieve microscale local photoactuation with satisfactory precision and amplitude.

**Figure 1:**
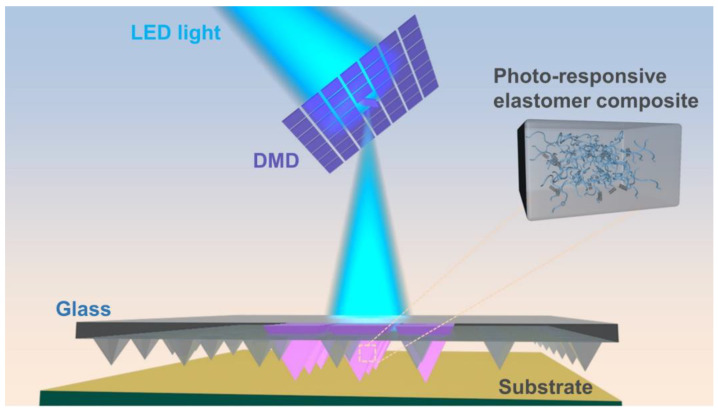
Scheme illustrating localized photoactuation for molecular printing. Digital micromirrors are used to direct light to selected pens for the nanolithography. The inset highlights the uniformly distributed functionalized short CNTs in PDMS.

In this communication, we prototype a simple yet potentially transformative strategy to achieve unprecedented local out-of-plane actuation desired for nanolithography (Figure 1). The progress is made possible by a fundamental understanding of the materials chemistry of the stimuli-responsive nanofillers, as well as the device innovation to deliver intense stimuli to microscale domains. On one hand, we synthesized and elucidated why the surface functionalized short carbon nanotubes (CNTs) are the optimal optically functional component in PDMS matrix through a comparative study. On the other hand, digital micromirror device (DMD) is applied to direct intense light locally for controlling the pens. We show that small-area local illumination (170 μm × 170 μm) can induce an out-of-plane motion of a very small group of pens (3 × 3) by exceeding 7 μm for photoactuated molecular printing, representing a three-orders-of-magnitude improvement in actuation resolution compared with the previous report [37].

## 2. Results and Discussion

**Figure 2:**
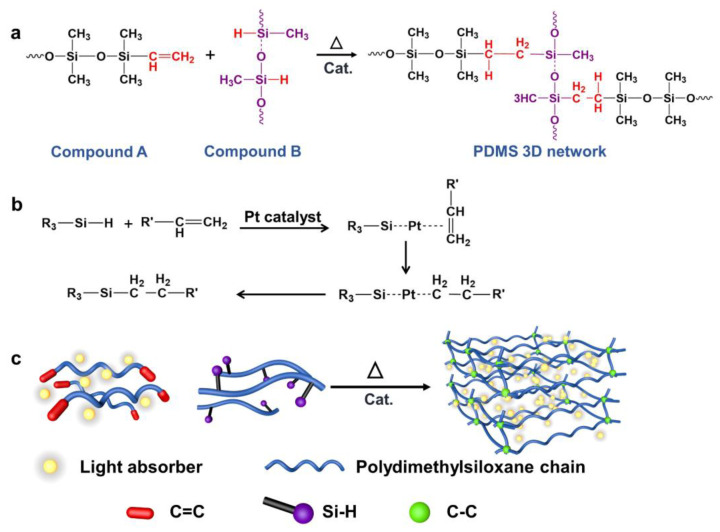
Reaction scheme elucidating the crosslinking chemistry of vinyl-terminated PDMS and the light absorbing PDMS composites. (**a**) Crosslinker (compound B)’s Si-H groups react with the vinyl groups of the prepolymer (compound A) to form a three-dimensional network. (**b**) General mechanism of platinum-catalyzed hydrosilylation for PDMS crosslinking. (**c**) Proposed synthetic procedures of the optically functional PDMS composite, with the nano fillers embedded in the prepolymer as the initial step. The vinyl terminal is labeled as the red cylinder.

We thermally crosslink silicones, instead of condensation polymerization or UV curing, [40,41] through the addition reaction between vinyl terminals (CH2=CH-) and silicon hydride (Si-H), due to the facile, non-toxic, and byproduct-free synthetic process. In such crosslinking reaction (as depicted in Figure 2a), the siloxane base oligomers (prepolymer) containing vinyl terminals are mixed with the base solution which mainly contains dimethylhydrogen siloxane, in the presence of a trace amount of Pt catalyst. The widely employed catalytic mechanism for Pt catalyst in hydrosilylation (illustrated in Figure 2b), [42,43] as initiated by Chalk and Harrod, [44] includes coordination to the alkene groups, oxidative addition of HSiR3, and the reductive elimination in the final step. As a result, multiple reaction sites on both the prepolymer and cross-linker promote 3D polymerization that can also be accelerated by heat. No waste product such as water or gas is generated during this reaction, which allows for molding with high spatial precision and reliable repeatability.

**Figure 3:**
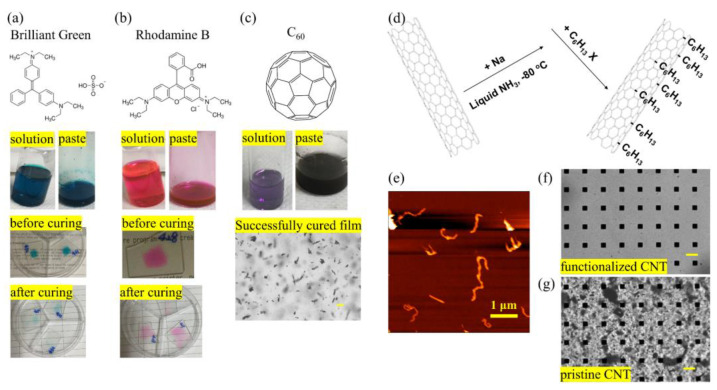
Colored PDMS composites containing various types of light absorbers: (**a**) Brilliant Green, (**b**) Rhodamine B, (**c**) C_60_, (**f**) functionalized CNTs, and (**g**) pristine CNTs. A general protocol with identical synthetic route was adopted, including solvent-assisted dispersion of the filler in the prepolymer, mixing with crosslinker after solvent removal, and thermal assisted crosslinking. The condition of the composite solution, paste, and film in these procedures are imaged and recorded. (**d**) Scheme showing surface grafting reaction of long alkyl chains (–(CH_2_)_5_CH_3_) on a CNT. (**e**) Atomic force microscopy image of functionalized short CNTs. The transmission optical microscope image of the composite pen arrays shows dramatic contrast on the uniformity of the light absorber distribution: (**f**) functionalized CNTs, (**g**) pristine CNTs. The scale bar represents 50 μm, unless specifically noted.

To fabricate light-absorbing elastomer composites (Figure 2c) for energy-efficient and spatially resolved photoactuation, we considered three key requirements for optical filler candidates. Firstly, the filler should be chemically inert and compatible with the PDMS crosslinking chemistry. Secondly, the filler should be uniformly embedded in PDMS matrix without significant aggregation, which is crucial to applications requiring film transparency. Thirdly, the photoactuation should show high energy conversion efficiency, which requires maximum photon absorption and least energy wasted through other processes such as fluorescence emission. We adopted multiple types of optical fillers and systematically examined the resulted PDMS-based composites. As shown in Figure 3 and Figure S1 in the supporting information (SI), typical organic dyes (such as Rhodamine B, Brilliant Green), carbon nanomaterials (such as C_60_ and its derivatives, carbon black, CNTs), and other inorganic light absorbers (such as MoS_2_) have been incorporated in PDMS polymer. For fair comparison, a general protocol with identical synthetic route was adopted (see the experimental section in Supplementary material), including: (i) solvent-assisted dispersion of the filler in the prepolymer, (ii) mixing with crosslinker after solvent removal, and (iii) thermal assisted crosslinking.

We found that the composite pastes containing Brilliant Green (Figure 3a) or Rhodamine B (Figure 3b) failed to cure. Instead, the paste spread around, and the color faded. We attribute this result to the following reasons. On the one hand, Si-H can be consumed, being added onto other unsaturated bonds in the molecular structure of the organic molecules, such as alkenes, alkynes, imines, and carbonyls [45,46]. On the other hand, lone pairs of certain nitrogen or sulfur-containing moieties tend to complex with and defunctionalize the Pt catalyst [47,48]. For example, the amine and sulfate group in Brilliant Green and the amine and carboxylic acid group in Rhodamine B are likely to interfere with the Si-H and the Pt catalyst and cause the failure of the crosslinking. Different from the organic dyes, inorganic light absorbers normally do not disrupt polymer curing; however, they suffer from agglomeration that leads to large-size aggregates. For example, a clear purple solution of C60 (Figure 3c) was obtained when mixed with PDMS prepolymer in toluene; however, large-size black trunks (indicating severe agglomeration) were observed after solvent removal, due to the drying kinetics and strong van der Waals interaction between the C60 molecules [49,50]. For carbon black (Figure S1a) or MoS2 nanopowders (Figure S1b), the result was even worse. These nanopowder fillers were insoluble in organic solvents, nor did they disperse well in the cured film. For the fullerene derivative [60]PCB-C8 (Figure S1c), clear red-brownish solution was obtained when mixed with PDMS prepolymer in chloroform; however, the paste could not cure in the final step, probably due to the reactive ester group in the molecular tail.

Among all the eight candidates we examined, the only nano filler that met the requirements is surface functionalized short CNTs. CNTs are attractive light absorbers that effectively couple optical, electrical, and mechanical properties in a single component, and surface grafting of long alkyl chains (–(CH_2_)_5_CH_3_) (Figure 3d) significantly enhanced their dispersion in the PDMS matrix. Short length of CNTs brings in double merits: lowering dispersion difficulty and preventing thermal percolation. The average length of the functionalized short CNTs was 435 nm (through analyzing 260 tubes using atomic force microscopy, Figure 3e). Consequently, a uniform and transparent film was fabricated (see the transmission optical microscopic image shown in Figure 3f and Figure S2a in SI). The control experiment showed that pristine, unfunctionalized CNTs formed large-size trunks in the PDMS matrix that cause substantial light scattering (Figure 3g). From these results, we can draw the following conclusions. First, the Si-H moiety in the PDMS crosslinker, in the presence of the Pt catalyst, can add to multiple bonds in the organic chromophores, resulting in the discoloration of the chromophores and failure in crosslinking. Secondly, the interactions between the filler units and that at the filler-polymer matrix interface dictate the dispersion condition of the light absorber in PDMS. The strong π-π stacking of the organic dyes or carbon nanomaterials causes parasite agglomeration or unwanted crystalline morphology, which prevents from forming transparent and uniform composite films. Therefore, the PDMS composite containing 0.25 wt% functionalized short CNTs was used to fabricate arrays of pyramid pens (at 60 or 100 μm-pitch over a 130 μm-thick backing layer, see Figure S2b,c in SI) for nanolithography applications.

**Figure 4:**
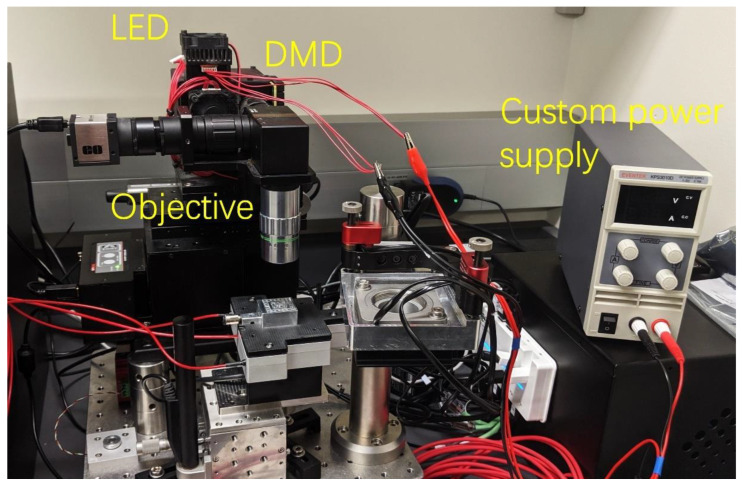
Photograph of the molecular printer featuring local high-power illumination capability. Key components are labeled, including the LED, DMD mirror, objective and a custom power supply supporting high-current operation of the LED.

Next, we demonstrate strong local photoactuation capable of realizing high-resolution photoactuated polymer pen lithography at the few-pen level. We built a DMD-integrated molecular printer with unprecedented capabilities to deliver high power illumination to microscale areas and active local administration (Figure 4). We note DMD has been utilized for spatially controlled photochemical patterning in massively multiplexed beam pen lithography [51,52]; however, it has not been applied to achieve local mechanical actuation. Several key innovations are made to convert the conventional DMD from a weak light deliver (~1.2 W cm^−2^ irradiation in 405 nm in Tera-fab E series printer) into a powerful photoactuation system. First, the DMD board was redesigned to house a heatsink that allows operation at high light intensities. Secondly, the illumination path between the light emitting diode (LED) and the DMD chip was shortened to reduce power losses from the uncollimated light. Thirdly, a custom power supply was used to run LED light source at a high current. These modifications enable the printer to project high-intensity visible light (405 nm in wavelength, ~4.5 W cm^−2^ at 20× objective) to selected areas in μm scale, without damaging the LED or DMD mirrors. In addition, each of the 786,432 micromirrors can be turned ON or OFF by computer, ensuring fully programmable photon delivery for active photoactuation of the composite pens within the array.

**Figure 5:**
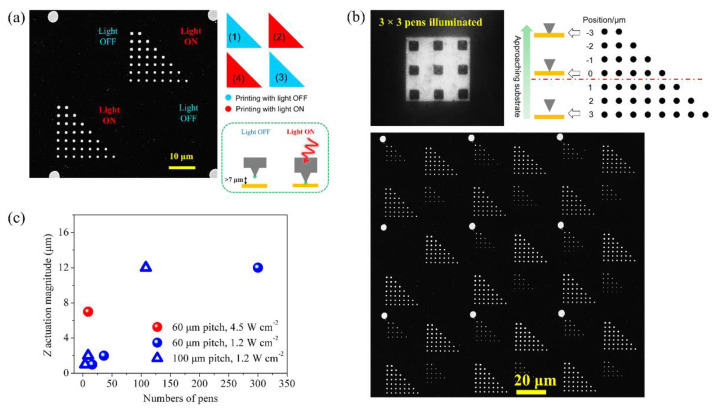
Localized photoactuation of the composite pens for molecular patterning. Visible light at 4.5 W cm^−2^ was used to drive 3 × 3 pens within a 2D array to engage the “paper” for active writing. (**a**) Scanning electron microscope (SEM) image of a representative pattern written by one of the illuminated pens. (**b**) SEM image of the pattern written by the illuminated pens. (**c**) The effect of the light intensity, illumination area, and the pitch of the pen array on the actuation resolution and magnitude.

With this powerful stimuli-delivery system, we further demonstrate that high-resolution local photoactuation enables active molecular printing with polymer pens. As a proof-of-concept patterning experiment, we printed features of 16-mercaptohexadecanoic acid (MHA) dots on Au-coated Si substrates using the fabricated pens. The vertical position *z*-dependent patterning experiment required to write seven rows of dots at programmed various pen-substrate distances (outlined by different *z* piezo extension values with vertical step of 1 μm between adjacent lines) in each triangle pattern. The pens were set to print four triangle arrays of dots: the second and fourth were printed under irradiation (red triangles schematically shown in the inset of Figure 5a), while the first and third were in the dark (blue triangles, Figure 5a). The dwell time was 4 s for writing each point. It should be noted that the light was incident to the selected area only during the dwell time (when writing the second and fourth triangle arrays), and the pens were programmed to cool for 10 s after writing each point to avoid any heat accumulation. We found when the light with 4.5 W cm^−2^ intensity was projected on an area of 170 μm × 170 μm (covering 3 × 3 pens with 60 μm-pitch), the illuminated pens experienced significant movement in *z* direction. A representative pattern written by one of the illuminated pens is displayed in Figure 5a. Seven lines of the dots could be written upon illumination, while in the dark no molecules were written. This observation unambiguously proves that the writing behavior can be solely controlled by light, and the out-of-plane movement of the illuminated pen at *z* direction is greater than 7 μm. A representative pattern written by the nine illuminated pens is displayed in Figure 5b (*z* was set as -3 to 3 μm, in which *z* = 0 was defined as the contact position where the pen was just engaged onto the printing substrate and ink delivery became possible). Similar photoactuation behavior was observed for all the illuminated pens. Compared to the patterns printed in the dark, three more lines of the dots could be written upon illumination. Additionally, the size of the dots written upon illumination was clearly larger, indicating that the pen was heavily pressed against the substrate. In fact, the average diameter of dots written at *z* = 3 under illumination was 1.03 μm, which is apparently larger than that (i.e., 730 nm) written in dark at *z* = -3. This indicates that the photoactuation resulted in an equivalent vertical movement of the pens by exceeding 6 μm, which agrees well with our findings. We also systematically examined the light actuating capability by varying the illumination intensity, illumination area, and the pitch of the pen array, and the result is summarized in Figure 5c. There are several important findings worth discussion. First, the actuation magnitude is positively correlated with the illumination area (i.e., the number of the light-covered pens). Secondly, the actuation magnitude depends on the illumination intensity, which agrees well with the linear relationship discovered previously [37]. Thirdly, the pitch between neighboring pens is inversely correlated with the number of the pens that can be actuated given a fixed illumination area, which is as expected.

The result in this work is significant and represents a major step towards realizing an individually actuated pen array. As shown in Table 1, resistive heaters can be embedded beneath the elastomeric backing layer to actuate a small group of pens via Joule heating [53]; however, the method is not scalable due to the difficulty in making independent electrical connections to individual pens when considering arrays of thousands or even millions of pens. Photoactuation does not need the integration of sophisticated physical systems because of its inherent advantage of wireless remote control. In this work, we demonstrate a key breakthrough to direct spatially selective photoactuation with micro-scale resolution in a programmable manner, which helps to overcome the vital challenge in selective photoactuation. Since 170 μm is in the pitch scale of the normal PPL design, it is possible to further reduce the number of pens (down to 1) in this illumination area by enhancing pitch length. This work lays the important foundation and indicates that the ultimate goal of single-pen actuation is within reach.

Photoactuated printing is analogous to tuning the *z* position of the pen in normal polymer pen lithography (PPL). Since the photoactuated expansion in *z* direction can be thoroughly and quantitatively documented, the resolution and precision of our printing technique in *x-y* and *z* direction should be similar to those of normal PPL. The capability of PPL to generate features with sub-100 nm resolution has been well established [36]. As for precision, for features made with one tip one can place them with sub-nm precision, but for tip-to-tip registry the precision should be closer to 100 nm because of the variation of the pens and can be further improved with high-quality Si masters.

## 3. Conclusions

In conclusion, we have demonstrated that unprecedented local photoactuation with high spatial resolution can be achieved through judicial choices of the light absorbing filler and innovation in local stimuli-delivery strategies. DMD was used, for the first time, to direct intense light to microscale domains for localized photoactuation. We find that PDMS and surface-functionalized short CNT nanofillers impart the composite with high uniformity and optical transparency. Together, these advances enable a 7 μm out-of-plane motion induced locally within a small area (170 μm × 170 μm), which represents over 5% strain of the supporting composite film, and a striking three-order-of-magnitude improvement in actuation resolution over the state of the art. This work marks a step towards the ambitious goal of active actuation at single-pen resolution for nanolithography [54,55]. Besides the further advance in microscopic light pattern delivery system, it is also possible to innovate novel pen architecture to better regulate the locally received energy, which can result in an enhanced energy utilizing efficiency and suppressed crosstalk. In addition, CNTs have stood out as exceptional ink ingredients or functional substrates in recent 3D printing and patterning research, [56,57,58,59] while our work reveals their application potential as dynamic lithographic tools.

## Figures and Tables

**Table 1:** Summary of the strategies for the dynamic actuation of polymer pens.

Actuation Approach	Typical Actuation Resolution	Actuation Magnitude (μm)	Potential for Scaling up	Year/Ref
resistive heating	4 × 4	2–4	challenge in fabricating complex system	2013 [53]
photoactuation through microscopic light	~3000	3	inability to address small groups of or individual pens	2018 [37]
photoactuation through DMD	3 × 3	>7	promising for scaling up	this work

## Data Availability

The data are available in the manuscript and Supplementary Materials.

## Supplementary Materials

The following supporting information can be downloaded at: https://www.mdpi.com/article/10.3390/molecules28031171/s1. The experimental section for synthesis of light absorbing PDMS composites, fabrication of composite pen arrays, and molecular printing with photoactuated pens. Figure S1: PDMS composite films with various light absorbers; Figure S2: images of the film and pen arrays made of PDMS and 0.25 wt% functionalized short CNTs [36,60,61].

## Sample Availability

Samples of the compounds are available from the authors.

## References

[1] Li M., Pal A., Aghakhani A., PenaFrancesch A., Sitti M. (2022). Soft actuators for real-world applications. Nat. Rev. Mater..

[2] Miriyev A., Stack K., Lipson H. (2017). Soft material for soft actuators. Nat. Commun..

[3] Rus D., Tolley M.T. (2015). Design, fabrication and control of soft robots. Nature.

[4] McEvoy M.A., Correll N. (2015). Materials that couple sensing, actuation, computation, and communication. Science.

[5] Mather P.T. (2007). Soft answers for hard problems. Nat. Mater..

[6] Lendlein A., Gould O.E.C. (2019). Reprogrammable recovery and actuation behaviour of shape-memory polymers. Nat. Rev. Mater..

[7] Ge F., Lu X., Xiang J., Tong X., Zhao Y. (2017). An optical actuator based on gold-nanoparticle-containing temperature-memory semicrystalline polymers. Angew. Chem. Int. Ed..

[8] Mahato M., Tabassian R., Nguyen V.H., Oh S., Nam S., Kim K.J., Oh I.-K. (2020). Sulfur- and nitrogen-rich porous π-conjugated COFs as stable electrode materials for electro-ionic soft actuators. Adv. Funct. Mater..

[9] Kim H., Ahn S., Mackie D.M., Kwon J., Kim S.H., Choi C., Moon Y.H., Lee H.B., Ko S.H. (2020). Shape morphing smart 3D actuator materials for micro soft robot. Mater. Today.

[10] Mura S., Nicolas J., Couvreur P. (2013). Stimuli-responsive nanocarriers for drug delivery. Nat. Mater..

[11] Ohm C., Brehmer M., Zentel R. (2010). Liquid crystalline elastomers as actuators and sensors. Adv. Mater..

[12] Ding T., Valev V.K., Salmon A.R., Forman C.J., Smoukov S.K., Scherman O.A., Frenkel D., Baumberg J.J. (2016). Light-induced actuating nanotransducers. Proc. Natl. Acad. Sci. USA.

[13] Matsubara K., Tachibana D., Matsuda R., Onoe H., Fuchiwaki O., Ota H. (2020). Hydrogel actuator with a built-in stimulator using liquid metal for local control. Adv. Intell. Syst..

[14] Lauback S., Mattioli K.R., Marras A.E., Armstrong M., Rudibaugh T.P., Sooryakumar R., Castro C.E. (2018). Real-time magnetic actuation of DNA nanodevices via modular integration with stiff micro-levers. Nat. Commun..

[15] Guo Y., Zhang J., Hu W., Khan M.T.A., Sitti M. (2021). Shape-programmable liquid crystal elastomer structures with arbitrary three-dimensional director fields and geometries. Nat. Commun..

[16] Kanygin M., Joy A.P., Bahreyni B. (2019). Localized mechanical actuation using pn junctions. Sci. Rep..

[17] Hwang I., Kim H.J., Mun S., Yun S., Kang T.J. (2021). A light-driven vibrotactile actuator with a polymer bimorph film for localized haptic rendering. ACS Appl. Mater. Interfaces.

[18] Koleoso M., Feng X., Xue Y., Li Q., Munshi T., Chen X. (2020). Micro/nanoscale magnetic robots for biomedical applications. Mater. Today Bio.

[19] Hedayati R., Mirzaali M.J., Vergani L., Zadpoor A.A. (2018). Action-at-a-distance metamaterials: Distributed local actuation through far-field global forces. APL Mater..

[20] Soto F., Wang J., Ahmed R., Demirci U. (2020). Medical micro/nanorobots in precision medicine. Adv. Sci..

[21] Bao B., Rivkin B., Akbar F., Karnaushenko D.D., Bandari V.K., Teuerle L., Becker C., Baunack S., Karnaushenko D., Schmidt O.G. (2021). Digital electrochemistry for on-chip heterogeneous material integration. Adv. Mater..

[22] Chowdhury S., Johnson B.V., Jing W., Cappelleri D.J. (2017). Designing local magnetic fields and path planning for independent actuation of multiple mobile microrobots. J. Micro-Bio Robot..

[23] Leroy E., Hinchet R., Shea H. (2020). Multimode hydraulically amplified electrostatic actuators for wearable haptics. Adv. Mater..

[24] Chen Y., Zhang Y., Karnaushenko D., Chen L., Hao J., Ding F., Schmidt O.G. (2017). Addressable and color-tunable piezophotonic light-emitting stripes. Adv. Mater..

[25] Whitesides G.M. (2006). The origins and the future of microfluidics. Nature.

[26] Schneider F., Draheim J., Kamberger R., Wallrabe U. (2009). Process and material properties of polydimethylsiloxane (PDMS) for optical MEMS. Sens. Actuator A Phys..

[27] McDonald J.C., Whitesides G.M. (2002). Poly(dimethylsiloxane) as a material for fabricating microfluidic devices. Acc. Chem. Res.

[28] Eduok U., Faye O., Szpunar J. (2017). Recent developments and applications of protective silicone coatings: A review of PDMS functional materials. Prog. Org. Coat.

[29] Zhou L., Song H., Liang J., Singer M., Zhou M., Stegenburgs E., Zhang N., Xu C., Ng T., Yu Z. (2019). A polydimethylsiloxane-coated metal structure for all-day radiative cooling. Nat. Sustain..

[30] Huh D., Kim H.J., Fraser J.P., Shea D.E., Khan M., Bahinski A., Hamilton G.A., Ingber D.E. (2013). Microfabrication of human organs-on-chips. Nat. Protoc..

[31] Cacucciolo V., Shintake J., Kuwajima Y., Maeda S., Floreano D., Shea H. (2019). Stretchable pumps for soft machines. Nature.

[32] Qin D., Xia Y., Whitesides G.M. (2010). Soft lithography for micro- and nanoscale patterning. Nat. Protoc..

[33] Wolf M.P., Salieb-Beugelaar G.B., Hunziker P. (2018). PDMS with designer functionalities—Properties, modifications strategies, and applications. Prog. Polym. Sci..

[34] Noimark S., Colchester R.J., Poduval R.K., Maneas E., Alles E.J., Zhao T., Zhang E.Z., Ashworth M., Tsolaki E., Chester A.H. (2018). Polydimethylsiloxane composites for optical ultrasound generation and multimodality imaging. Adv. Funct. Mater..

[35] Ajayan P., Tour J. (2007). Materials science—Nanotube composites. Nature.

[36] Eichelsdoerfer D.J., Liao X., Cabezas M.D., Morris W., Radha B., Brown K.A., Giam L.R., Braunschweig A.B., Mirkin C.A. (2013). Large-area molecular patterning with polymer pen lithography. Nat. Protoc..

[37] Huang Z., Li L., Zhang X.A., Alsharif N., Wu X., Peng Z., Cheng X., Wang P., Brown K.A., Wang Y. (2018). Photoactuated pens for molecular printing. Adv. Mater..

[38] Horiuchi N. (2018). Photoactuated printing. Nat. Photonics.

[39] Li L., Huang Z., Wang Y., Brown K.A. (2019). Design of elastomer-CNT film photoactuators for nanolithography. Polymers.

[40] Choi K.M., Rogers J.A. (2003). A photocurable poly(dimethylsiloxane) chemistry designed for soft lithographic molding and printing in the nanometer regime. J. Am. Chem. Soc..

[41] Bhattacharjee N., Parra-Cabrera C., Kim Y.T., Kuo A.P., Folch A. (2018). Desktop-stereolithography 3D-printing of a poly(dimethylsiloxane)-based material with sylgard-184 properties. Adv. Mater..

[42] Troegel D., Stohrer J. (2011). Recent advances and actual challenges in late transition metal catalyzed hydrosilylation of olefins from an industrial point of view. Coordin. Chem. Rev..

[43] Meister T.K., Riener K., Gigler P., Stohrer J., Herrmann W.A., Kühn F.E. (2016). Platinum catalysis revisited-unraveling -principles of catalytic olefin hydrosilylation. ACS Catal..

[44] Chalk A.J., Harrod J.F. (1965). Homogeneous catalysis. II. the mechanism of the hydrosilation of olefins catalyzed by group VIII metal complexes1. J. Am. Chem. Soc..

[45] Marciniec B. (1992). Comprehensive Handbook on Hydrosilylation.

[46] Marciniec B., Maciejewski H., Pietraszuk C., Pawluc P. (2009). Hydrosilylation: A Comprehensive Review on Recent Advances.

[47] Product Information about Dow Corning Brand Silicone Encapsulants. http://bdml.stanford.edu/twiki/pub/Rise/PDMSProceSS/PDMSdatasheet.pdf.

[48] Parbhoo B., O’Hare L.A., Leadley S.R., Dillard D.A., Pocius A.V., Chaudhury M. (2002). Chapter 14—Fundamental aspects of adhesion technology in silicones. Adhesion Science and Engineering.

[49] Astefanei A., Núñez O., Galceran M.T. (2015). Characterisation and determination of fullerenes: A critical review. Anal. Chim. Acta.

[50] Bogdanov A.A. (2020). Processes of aggregation of fullerene C_60_ in polymer–fullerene composites. Phys. Solid State.

[51] Liao X., Brown K.A., Schmucker A.L., Liu G., He S., Shim W., Mirkin C.A. (2013). Desktop nanofabrication with massively multiplexed beam pen lithography. Nat. Commun..

[52] Carbonell C., Valles D.J., Wong A.M., Tsui M.W., Niang M., Braunschweig A.B. (2018). Massively multiplexed tip-based photochemical lithography under continuous capillary flow. Chem.

[53] Brown K.A., Eichelsdoerfer D.J., Shim W., Rasin B., Radha B., Liao X., Schmucker A.L., Liu G., Mirkin C.A. (2013). A cantilever-free approach to dot-matrix nanoprinting. Proc. Natl. Acad. Sci. USA.

[54] Salaita K., Wang Y., Fragala J., Vega R.A., Liu C., Mirkin C.A. (2006). Massively parallel dip–pen nanolithography with 55 000-pen two-dimensional arrays. Angew. Chem. Int. Ed..

[55] Salaita K., Wang Y., Mirkin C.A. (2007). Applications of dip-pen nanolithography. Nat. Nanotech..

[56] Gallastegui A., Dominguez-Alfaro A., Lezama L., Alegret N., Prato M., Gómez M.L., Mecerreyes D. (2022). Fast visible-light photopolymerization in the presence of multiwalled carbon nanotubes: Toward 3D printing conducting nanocomposites. ACS Macro Lett..

[57] Zhu Y., Ramadani E., Egap E. (2021). Thiol ligand capped quantum dot as an efficient and oxygen tolerance photoinitiator for aqueous phase radical polymerization and 3D printing under visible light. Polym. Chem..

[58] Wang P., Barnes B., Huang Z., Wang Z., Zheng M., Wang Y. (2021). Beyond color: The new carbon ink. Adv. Mater..

[59] Huang Z., Powell L.R., Wu X., Kim M., Qu H., Wang P., Fortner J.L., Xu B., Ng A.L., Wang Y. (2020). Photolithographic patterning of organic color-centers. Adv. Mater..

[60] Deng S., Zhang Y., Brozena A.H., Mayes M.L., Banerjee P., Chiou W.-A., Rubloff G.W., Schatz G.C., Wang Y. (2011). Confined propagation of covalent chemical reactions on single-walled carbon nanotubes. Nat. Commun..

[61] Huo F., Zheng Z., Zheng G., Giam L.R., Zhang H., Mirkin C.A. (2008). Polymer Pen Lithography. Science.

