# Supplementary material for: Localized Photoactuation of Polymer Pens for Nanolithography
Source: Molecules. 2023 Jan 25;28(3):1171. doi: 10.3390/molecules28031171 (PMC9919257; doi:10.3390/molecules28031171)
Supplement: Supplementary file 1 [file molecules-28-01171-s001.zip › molecules-2179762-supplementary.pdf]

Supplementary Materials

Localized photoactuation of polymer pens for nanolithography

Zhongjie Huang,[1,*] Shaopeng Li,[1] Jiaqi Zhang,[1] Huan Pang,[2] Andrey Ivankin,[3] YuHuang Wang[4,5,*]

1. State Key Laboratory for Modification of Chemical Fibers and Polymer Materials, College of Materials Science and Engineering, Donghua University, Shanghai, 201620, China

2. School of Chemistry and Chemical Engineering, Yangzhou University, Yangzhou, 225009, China

3. TERA-print, LLC, 8045 Lamon Ave, Suite 330, Skokie, IL 60077, USA

4. Department of Chemistry and Biochemistry, University of Maryland, College Park, MD 20742, USA

5. Maryland NanoCenter, University of Maryland, College Park, MD 20742, USA

* Correspondence: huangzhongjie@dhu.edu.cn (Z.H.); yhw@umd.edu (Y.W.)

Experimental Section

Synthesis of light absorbing PDMS composites. Organic dyes (Rhodamine B, Brilliant Green), carbon nanomaterials (C_{60}, [60]PCB-C8, carbon black, CNTs) and other inorganic light absorbers (MoS_2) were incorporated in PDMS composites. For fair comparison, a general protocol with identical synthetic route was adopted. First, the light absorber and PDMS precursor (Sylgard 184, Dow Corning) were dissolved or dispersed in organic solvents (such as chloroform or toluene), and the solvent was then removed. The paste was mixed thoroughly with the crosslinker (1/5 of the mass of the prepolymer) and the composite film was fabricated by doctor-blading the paste on the glass slide, and cured at 80 ^{o}C for 4 hrs afterwards. For functionalized CNTs, CNTs (SWeNT® SMW 100) were covalently grafted with long alkyl chains $–(CH_2)_5CH_3$ using Billups-Birch alkylation.[60] The functionalized CNTs were characterized using a Cypher ES AFM (Asylum Research Corporation). The lengths of ~260 CNTs were analyzed and recorded.

Fabrication of composite pen arrays. The pen arrays were fabricated following a previously reported protocol.[36,61] The Si master (TERA-print LLC) included an array of pyramidal holes (with a pyramid base length of 20 μm and pitch distance of 60 or 100 μm), and was surface-treated with the fluorinated silane reagent (heptadecafluoro-1,1,2,2-tetra(hydrodecyl)trichlorosilane) for easy release. The composite paste (PDMS with 0.25 wt% of functionalized short CNTs) was

dropped on top of the Si master, and gently pressed by a superflat glass slide (4 cm × 4 cm, CG-1904-36, ChemGlass). The pen arrays were cured at 80 ^{o}C for 12 hrs, allowing thorough crosslinking. The backing layer thickness of the pen array was measured by a profilometer (Tencor Alpha Step 200). The edges and corners of the pen array were deposited with 5 nm of Cr and 100 nm of Au (see Figure S2c) in a thermal deposition system and served as the conductive electrodes for parallel alignment.

Molecular printing with photoactuated pens. The composite pens were applied for ink patterning using a custom-built molecular printer based on TERA Fab E-series system (see Figure 4 in the manuscript). The modification included the redesigned DMD board to house a heatsink, the shortened illumination path between the LED and the DMD chip, and a custom power supply to run LED at a higher intensity. As a result, the printer could project high-intensity visible light (405 nm in wavelength, ~4.5 W cm^{-2}) when a 20× objective (0.42 NA, Olympus) was used.

The classic molecular ink of 5 mM ethanolic solution of 16-mercaptohexadecanoic acid (MHA) was loaded on the pen arrays before patterning experiments. Si <100> wafers were thermally deposited with 5 nm of Cr and 35 nm of Au, and diced into chips with the size of 2 cm × 2 cm for use as patterning substrates. The patterning environment was maintained at room temperature. The vertical position z-dependent patterning experiment required to write seven rows of dots at programmed various pen-substrate

distances (outlined by different *z* piezo extension values with vertical step of 1 μm between adjacent lines) in each triangle pattern. The pens were set to print four triangle arrays of dots: the second and fourth were printed under irradiation, while the first and third in the dark. The dwell time was 4 s for writing each point. The light was incident to the selected area only during the dwell time (when writing the second and fourth triangle arrays), and the pens were programmed to cool for 10 s after writing each point to avoid any heat accumulation. The effect of the light intensity, illumination area and the pitch of the pen array on the actuation resolution and magnitude were systematically examined. After patterning, samples were etched for about 5 min in a freshly mixed aqueous solution containing 27 mM $Fe(NO_3)_3$ and 40 mM thiourea. The printed ink existed as a self-assembled monolayer on gold and protected the gold layer from etching. As etching, the remained patterns were examined by Hitachi SU-70 Schottky field emission gun scanning electron microscope with an accelerating voltage of 5.0 kV.

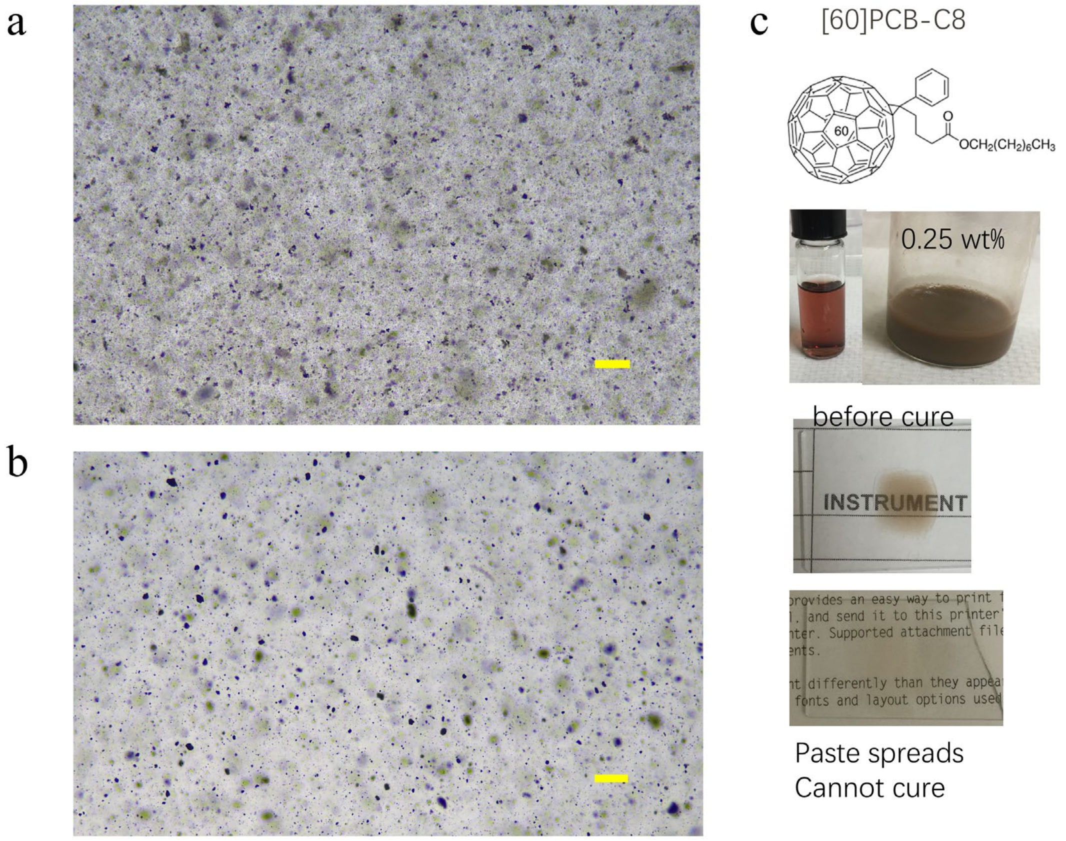

Figure S1. PDMS composite films with various light absorbers: (a) carbon black, (b) MoS_2, (c) fullerene derivative [60]PCB-C8. Transmission optical image of the composite film with (a) carbon black, (b) MoS_2. The scale bar represents 50 μm. (c) The image of the PDMS based composite solution (in chloroform), paste and film with 0.25 wt% of fullerene derivative [60]PCB-C8 (molecular structure shown as the inset). It turned out that the paste could not cure and spread around instead.

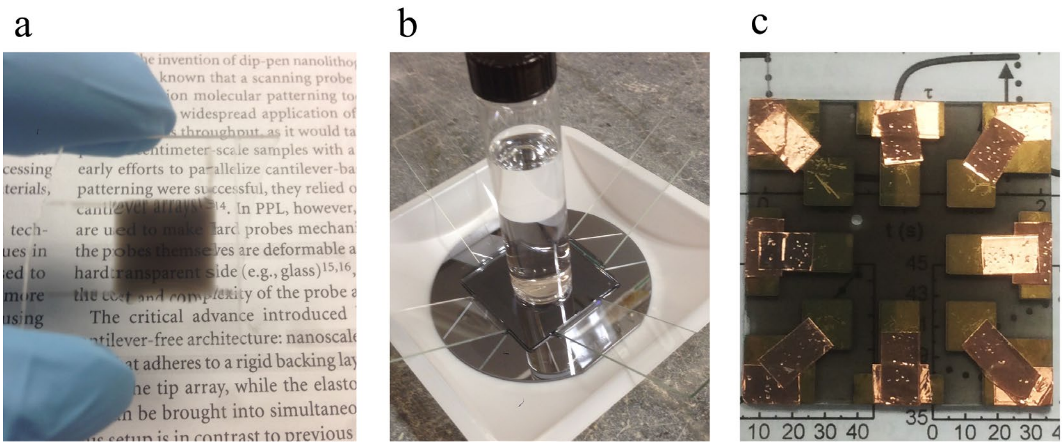

Figure S2. Images of the film and pen arrays made of PDMS and 0.25 wt% functionalized short CNTs. (a) Image of the uniform film on glass slide, showing panchromatic light absorption with high optical transparency. (b) In the molding process, the composite paste was dropped on top of the Si master, and a superflat glass slide (4 cm × 4 cm) was placed on it and pressed gently. (c) Image of the resulted pen array. The edges and corners of the pen array were thermally deposited with 5 nm of Cr and 100 nm of Au and served as the conductive electrodes for parallel alignment before printing.
